# Safe management of full-capacity live/mass events in COVID-19 will require mathematical, epidemiological and economic modelling

**DOI:** 10.1177/01410768211007759

**Published:** 2021-04-19

**Authors:** M Harris, J Kreindler, A El-Osta, T Esko, A Majeed

**Affiliations:** 1 Department of Primary Care and Public Health, Imperial College London, London W6 8RP, UK; 2 Institute of Genomics, 37546 University of Tartu is Riia 23b, 51010, Tartu, Tartumaa, Estonia

The importance of the live events industry to the UK economy is significant, with the creative industries^1^ alone contributing £117bn to the UK economy in 2018.^1^ However, the public health response to COVID-19 on various sectors of the UK economy led to an unprecedented fall in theatrical sales of 93%,^2^ with the entertainment industry estimated to lose £110 m per month of full closure.^3^ Several high-profile live music events have been cancelled.^4,5^ There has been limited experience of the reopening of live events in other countries^6^; however, this has only been possible due to effective public health interventions to reduce community transmission to near zero levels. The sustainability of stringent border control measures to virus transmission is much debated; however, it is clear that the ability for the UK to achieve and then sustain low community transmission levels will require rigorously monitored borders and quarantine measures for inbound travellers. Widespread population immunity through vaccination (and from previous infection) will help the UK to reach low transmission levels; however, the success of the vaccine programme will largely depend on convergent evolution of the virus but this remains unknown. Additional measures to stringent social distancing, isolating at home and high uptake of the vaccination programme to achieve herd immunity to existing and emergent mutant strains of coronavirus will all be required to maintain low transmission levels in the UK. However, because of vaccine hesitancy among some groups, there may be areas of the UK where COVID-19 outbreaks continue.

Mass events such as live music concerts, festivals, congresses, theatrical events and educational conferences are not considered essential businesses. Because the experience requires people to be very close together, live events generally create conditions that favour virus transmission. Indoor events are much more likely to worsen transmission than outdoor events due largely to enhanced exposure to aerosols. The economics of the live entertainment industry requires operating to near 100% capacity to be profitable for the live event organisers. In reality, even operating at 60% capacity after reopening will lead to 6.8 m fewer event admissions, causing a reduction in £255 m in revenue over six months^3^; but even this is optimistic since adherence to the strict 2 -m social distancing rule would reduce capacity by more than half. Policy options will be required that balance the risk to individuals and public health, while also permitting the industry to reopen.

As first addressed by Melvin Benn in the LiveNation Full Capacity Plan,^7^ there are currently no policy prescriptions, systems, protocols or practices in place to permit the return of live entertainment at full capacity without putting people and the health system at risk by increasing the likelihood of super-spreading events. Certain self-care and risk mitigation strategies such as including wearing facemasks, handwashing, and social distancing and lockdowns are the only current non-pharmaceutical interventions available to reduce the basic reproduction rate of the virus. Despite the early successes of the UK vaccination programme, increased uptake and coverage alone will not guarantee elimination of ongoing transmission or the emergence of new mutant strains. Even assuming herd immunity acquired through vaccination or infection is possible, it might subsequently be eroded continuously as a result of viral mutation and waning immunity. Furthermore, the protracted timeframe expected to achieve this would result in the irrevocable collapse of the sector following attempts to remain solvent. Furthermore, because vaccines are not currently licensed for children and many people are reluctant to receive the vaccine at all, this could lead to a significant pool of susceptible individuals. Any solution that involves drawing on existing public health infrastructure to test asymptomatic people simply for the purposes of attending a live event is untenable and will likely prove uneconomical.

## Live/mass events and COVID-19 transmission

There have been various reports of notable super-spreading events identified following attendance at live events including a church event in Arkansas (estimated attack rate 38%–75%),^8^ as well as at a conference in South Korea where a single infected individual led to a cluster of around 5000 cases and a nightclub in Seoul where one individual infected 100 others.^9^ Avoidable super-spreading events play an important role in influencing the transmission dynamics of the pandemic.^9^ Super-spreading events are largely dependent on biological (i.e. viral load, susceptibility) and physical factors (i.e. size and ventilation of the venue), whereas singing and speaking loudly in particular have been described as an important factor, placing live events at particular risk.^9^ Unsurprisingly, there has been a complete moratorium on live events, and only are few studies have assessed the efficacy of different strategies to support the reopening of events at full capacity. Notably, the RESTART-19 study and the PRIMAVERA studies assessed transmission dynamics in a live concert, with PRIMAVERA providing on-the-door antigen testing, 1:1 randomised admittance with an eight-day follow-up testing. Both studies suggested that transmission could be reduced by improving ventilation in the venue, mandating the use of face masks, minimising social mixing and adherence to good hygiene practices. However, both studies were conducted at around 50% capacity, a level which is in most cases economically unsustainable.^10^ From 21 June 2021, live events in the UK are permitted, however not at full capacity.

## Pre-event home testing

As PRIMAVERA showed, pre-event testing is essential to ensure that only COVID-19-negative ticketholders enter the venue. This was achievable in the context of the trial because participants consented to testing, and were then randomised into entry to the venue, on the day of the event. However, there are profound ethical issues arising from sending positive cases (or false positives) and their close contacts home in a real-world setting, especially if ticketholders will be using public transport for the return journey and the associated risks of transmission to others. To address this, pre-event testing would need to be conducted in the home setting and with sufficient time in advance to ensure that ticket-holders who test positive are identified and signposted to get appropriate support and refunds prior to journeying to the live event. Polymerase Chain Reaction tests would not be universally viable for all events due to the expense and burden on laboratory infrastructure. Furthermore, reliance on PCR tests that take longer to process would widen the post-test pre-event window of opportunity for infection, and would further compromise the utility for any testing programme. The faster and significantly less costly lateral flow test^11^ which have been used routinely in sub-Saharan Africa and other low- and middle-income countries since the beginning of the pandemic makes this type of test more adequate for the purposes described. Whereas lateral flow tests are around 20% less accurate than PCR tests,^12^ most lateral flow tests have acceptable levels of sensitivity and specificity versus PCR albeit for symptomatic cases (e.g. Panbio COVID-19 Ag Rapid Test Device (Abbott GmBH) Sensitivity 93.3%/Specificity 99.4%; STANDARD Q COVID-19 Ag Test, SD Biosensor, Inc (Roche) Sensitivity 96.5%/Specificity 99.7%).^13^ Innova has been granted an Emergency Use Authorization for the UK National Testing Programme and versions of the above authorised for supervised remote self-testing are now available. Crucially, the overall performance of lateral flow tests is dependent on the collection of a valid sample. A mass home-testing initiative in Liverpool resulted in the significant reduction in test positivity rates primarily because of inadequate sample collection due to a lack of training when administering the test in the home setting.^13^ Recently, lateral flow tests that use saliva collection are being piloted^14^; however, whether this will mitigate the aforementioned issues has not yet been well-studied. Reliability and efficacy of the tests improves with training and in some real-world settings home based lateral flow tests have been shown to be reliable.^13^

## A business and operational model

The planned reopening of live events requires a congruent business and operational model that puts safety first, and that works for industry as well as individuals. For a model to be successful, the event organisers would need to sponsor, procure, manage and coordinate all testing and entry to the venue while adhering to extant public health safety measures to limit the spread or coronavirus. Given these conditions, a working model would need to be based on three pillars:

1. Use quality assured and streamlined pre-testing procedures to ensure that transmission remains no higher, and possibly lower than, background community levels.
2. Pre- and post-testing workflow should be secure, feasible, convenient and acceptable to both ticket holders and event organisers alike.
3. Real-time assessment of the risk based on data-led approaches that inform the ticketholder (and their household), the event organiser (and their local public health authority) and health systems (and government policy makers) should the event take place.

## A working model

**Figure 1.**
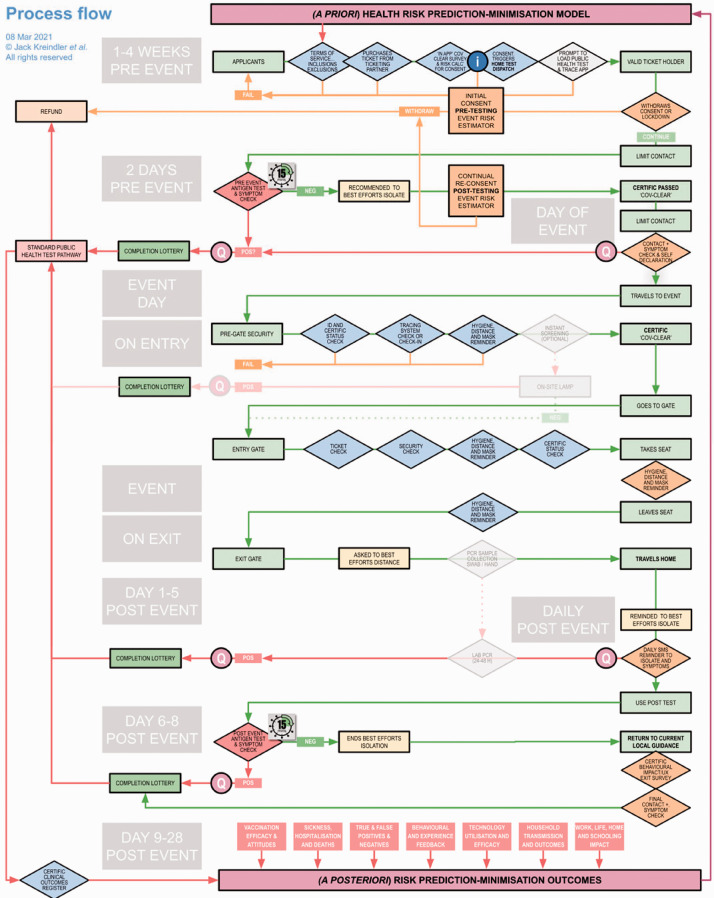
Detailed process map for a commercial-public health monitoring system to facilitate attendance at a live event to be as safe as community transmission.

We propose an infection and immunity testing and surveillance system that could facilitate the reopening of mass/live events (Figure 1). Briefly, once an event is announced, the customer would purchase a ticket to an event, which would become valid only after they complete a risk questionnaire and complete a home test. The test would be videoed or live streamed to a professionally trained testing control officer, allowing for near-real-time assessment validation of the patient and test kit identity, and validity of the test result depending on the assessment of the sample collection method. Ticket holders that show a positive result would be alerted and will quality for an automatic full refund of the ticket price and the corresponding ticket marked as invalid and notification given to public health authorities. Mass events, such as the Olympics, routinely use live streamed testing control officers for anti-doping measures and so the workforce and the technology is already in place to facilitate such processes. Tickets held by COVID-19-positive individuals can be released to geographies with lower prevalence to ensure full capacity at the event. Ticket holders with a negative test result would receive a privacy-preserving scannable (e.g. QR coded) certificate to show at the event entrance thereby gaining access to the event. At the event, the ticket holders would follow any feasible mitigations in a full capacity setting for distancing, mask-wearing and hand hygiene aimed to achieve the expected minimum transmission at the event. In trials, the ticket holder would recommended a five-day ‘best efforts’ self-isolation protocol and a follow-up home-based questionnaire daily and a test-to-release home test as per the pre-test process, and then released from self-isolation if a negative (valid) result is obtained and verified. There are opportunities for ticket holders to game such a system, particularly the chance of an eagerly anticipated live event is an incentive to mis-swab. However, rewarding positive adherence to testing protocols and honest submission of household data with an incentive, reward or lottery for future tickets may mitigate such risks. Furthermore, the provision of individual risk scores (see below) for informed consent to attend the event is improved through accurate data. It would be in the interests of the ticket holder to provide accurate information.

An inevitable concern that biometric data are kept in safe storage is non-trivial and will require several assurances. First, robust, independent academic oversight and ethics approval for data storage and sharing. Second, pseudonymised data storage and a clear separation between event organisers storing consumer purchasing data and third-party certificated testing and risk modelling services that will retain biometric data under strict confidentiality. This way, event organisers will have no access to individual biometric data other than to receive a go-no go signal from the risk modelling service in the form of a scannable QR code at entry to the event.

## Risk prediction model

Based on the pre-event test result, questionnaires, vaccine status and background prevalence, it would be possible to model the number and infectivity of individuals entering an event and the risk of transmission at the event which takes into account (1) journeying to the event, (2) in-venue point particle interactions at the event and (3) journeying back home from the event. Using Bayesian adaptation, the multi-component model can be iteratively refined each time there is an event to increase predictive accuracy. Input variables would include current community incidence, date of event and duration, number of ticket holders attending the event, size of the event venue and ventilation standards, time between test result and event, household close contacts and presence of underlying risk factors. Moreover, an open Application Programming Interface will allow the model to be shared across industry and for other events to contribute and improve the model.

Standard statistical algorithms can be ‘plugged in’ for the in-venue transmission likelihood based on the best available models. Before the event, a combination of the Jimenez Aerosol transmission model, based on the Wells-Riley equations, with Monte Carlo simulations will allow an estimation of the distribution of the probable number of cases, hospitalisations and deaths.^15^ The comparison of the outcomes of the simulations performed with or without various protocols could thus permit an estimation of their efficacy under different scenarios. The primary purpose of the model would not be to eliminate risk of transmission at the event because there will inevitably be some risk given some inaccuracy in the lateral flow tests. The model is therefore primarily aimed at minimising known risks down to background community-level transmission or even lower. Such a risk estimation engine will necessarily feature path dependencies, non-linear associations and feedback loops and there will need to be assumptions made. However, currently there is no possibility of estimating individual-level and event-level risk of transmission and so, acknowledging that they will need to continuously learn and improve, it is important to get the mechanisms in place for risk estimation. Some risk estimation may be better than no risk estimation. The government has signalled the importance of mass testing of event attendees in order for the safe, reopening of full capacity live events.^16^

## Conclusion

In this article, we propose a transmission monitoring system and reimbursement model that has the potential to support the live events industry to re-open at capacity while reducing the risk of transmission to equal or lower-than-community levels. Several steps need to be tested in the model, including acceptability to customers, the commercial viability to the live events industry and compliance with the post-event requirements. Different scenarios should be explored including the price point for ticket sales that would support a return to profitability in relation to the size and frequency of live events that can support such a system. However, as a model that potentially meets the needs of industry, consumers, the health system and public health, this collaborative approach merits testing. This will be of paramount importance not only to the entertainment industry but also to other mass events such as those for educational purposes and conferences. In future, ‘digital vaccine certificates’ will rationally modulate pre- and post-testing needs; however, this model offers a first step towards society learning to live with SARS-CoV-2 in a fully reopened economy.
